# Method: low-cost delivery of the cotton leaf crumple virus-induced gene silencing system

**DOI:** 10.1186/1746-4811-8-27

**Published:** 2012-08-01

**Authors:** John Richard Tuttle, Candace H Haigler, Dominique Robertson

**Affiliations:** 1 Department of Crop Science, North Carolina State University, Raleigh, NC 27695, USA; 2 Department of Plant Biology, North Carolina State University, Raleigh, NC 27695, USA

**Keywords:** Cotton, VIGS, Virus-induced gene silencing, Cotton leaf crumple virus, Agroinoculation, Particle inflow gun, Bombardment, Gossypium

## Abstract

**Background:**

We previously developed a virus-induced gene silencing (VIGS) vector for cotton from the bipartite geminivirus*Cotton leaf crumple virus* (CLCrV). The original CLCrV VIGS vector was designed for biolistic delivery by a gene gun. This prerequisite limited the use of the system to labs with access to biolistic equipment. Here we describe the adaptation of this system for delivery by *Agrobacterium *(*Agrobacterium tumefaciens*). We also describe the construction of two low-cost particle inflow guns.

**Results:**

The biolistic CLCrV vector was transferred into two *Agrobacterium * binary plasmids. Agroinoculation of the binary plasmids into cotton resulted in silencing and GFP expression comparable to the biolistic vector. Two homemade low-cost gene guns were used to successfully inoculate cotton (*G. hirsutum) * and *N. benthamiana* with either the CLCrV VIGS vector or the Tomato golden mosaic virus (TGMV) VIGS vector respectively.

**Conclusions:**

These innovations extend the versatility of CLCrV-based VIGS for analyzing gene function in cotton. The two low-cost gene guns make VIGS experiments affordable for both research and teaching labs by providing a working alternative to expensive commercial gene guns.

## Background

Virus-induced gene silencing (VIGS) is a reverse genetics technique that exploits the plant's post-transcriptional gene silencing (PTGS) machinery to obtain a sequence-specific transcript reduction for a targeted gene [1,2]. The technique employs a virus that has been modified to carry a fragment of a host gene. When the virus infects the plant, it triggers PTGS against the viral genome as well as the included host sequence. This leads to the degradation of RNAs with homology to the viral genome and produces a knockdown phenotype for the targeted gene(s). Because it can rapidly silence genes without the need for stable transformation, VIGS has become an attractive alternative to other reverse genetics strategies, which are time-consuming and especially difficult in plant species like cotton that are recalcitrant to transformation/regeneration.

VIGS vectors have been developed from a variety of virus/host combinations [1-3]. Although vectors have been constructed from RNA and DNA viruses as well as viral DNA satellites, we will only focus on vectors used in this research as derived from the bipartite DNA viruses of the family Geminiviridae, genus *Begomovirus*. These are single stranded DNA viruses with a conserved, well-characterized genome organization. The two genome components are designated DNA A and DNA B. Sequences coding for replication and movement proteins are split between the A and B components, respectively [4]. The A component contains 5 predicted open reading frames that code for the replication related proteins AL1 and AL3, the transactivator/anti-silencing protein AL2, the putative silencing suppressor AL4, and the coat protein AR1. The B component contains two open reading frames that code for the intercellular and intracellular movement proteins, BL1 and BR1 respectively. The two components share a ~200-bp region of high homology [4] referred to as the common region, which contains the origin of replication and a consensus sequence that is cleaved and ligated by the AL1 protein during rolling circle replication [5,6]. When a common region is placed on either side of the genome as a direct repeat, the region between the replication origins is released in planta to form a functional viral episome [7,8].

To accommodate targeting sequences, a multiple cloning site is typically inserted either in place of the *AR1* sequence or downstream of the *BR1* gene [9-12]. Peele and coworkers reported that the latter approach resulted in more extensive silencing, but insertion of sequences downstream of the CLCrV*BR1* gene failed to result in a systemic infection [9], [Tuttle and coworkers, unpublished]. The deletion or replacement of *AR1* sequence renders begomoviruses non-transmissable by their whitefly vector [13,14]. Therefore, to achieve VIGS the viral vector must be introduced into the plant cells either mechanically or through the use of *Agrobacterium * vectors.

Particle bombardment employs a “gene gun” to blast particles coated with viral DNA into the plant. The gene gun can be commercial (BioRad’s Helios or PDS-1000) or a homemade particle inflow gun (PIG; [15]). Both are powered by pressurized helium. A solenoid valve on the gas cylinder controls the rapid release of helium, which passes into a vacuum chamber through a filter holding micron or submicron particles of gold or tungsten that carry the nucleic acids. The metal particles are then forced into the samples below the filter [15]. Although there have been several publications detailing the construction of homemade gene guns, some degree of technical skill is required for their construction. This together with the high cost of commercial gene guns means that VIGS vectors that rely on biolistic delivery methods are not useful in all labs [15-17].

There are two other relatively easy and inexpensive methods for inoculating begomovirus vectors: DNA abrasion and agroinoculation. DNA abrasion involves the use of an abrasive (carborundum or ground glass) to introduce a viral DNA solution into leaf cells [18]. Although this method of inoculation is reasonably efficient for viruses that are not phloem-limited, it is not effective for all virus/host combinations, including Cotton leaf crumple virus (CLCrV)/cotton, the combination described here. The success of this method depends in part on the tissue specificity of the virus as well as the mechanical properties of the host leaf being inoculated [18,19]. For inoculation using *Agrobacterium *[20], similar to biolistic vectors, each component of the vector must be flanked by directly repeated common regions. For bipartite begomoviruses, a mixture of *Agrobacterium *carrying the A and B component plasmids is introduced into the plant through stem inoculation, wounding, or infiltration into intercellular leaf spaces [20-22]. In each of these methods, a single unit-length viral genome component is released from plasmid DNA to establish a systemic infection.

Cotton leaf crumple virus (CLCrV) is a cotton-infecting geminivirus endemic to the southwestern United States and Mexico [23]. Because it is a vascular-associated virus [12,24] it is more difficult to inoculate than other begomoviruses that show broader tissue specificity [19]. We developed the A DNA as a vector for foreign DNA by replacing the coat protein gene with a multiple cloning site and demonstrated VIGS in cotton (*Gossypium hirsutum*) [12]. Co-bombardment of cotyledons with the modified A DNA and wild type B-DNA produced systemic silencing in several *G. hirsutum* cultivars that persisted throughout the plant for over a year and was most extensive in the cultivars Acala SJ-1 and Deltatype Weber [25]. VIGS was visualized using a 500-bp fragment of the chlorophyll biosynthetic gene, *Magnesium Chelatase* subunit I (*ChlI*). Silencing of this gene produced a sectored pattern of chlorophyll loss that was more extensive at lower growth temperatures [12]. We also showed that CLCrV could be used as an expression vector by inserting a full-length GFP in place of the coat protein gene [12].

In this paper, we expand the potential of cotton VIGS by: (a) demonstrating the effectiveness of CLCrV VIGS vectors after moving them into binary vectors for agroinoculation and (b) providing instructions for making two inexpensive gene guns and demonstrating their potential for inoculating VIGS vectors.

## Results and discussion

### Binary vectors for *Agrobacterium *-mediated delivery of CLCrV

**Figure 1:**
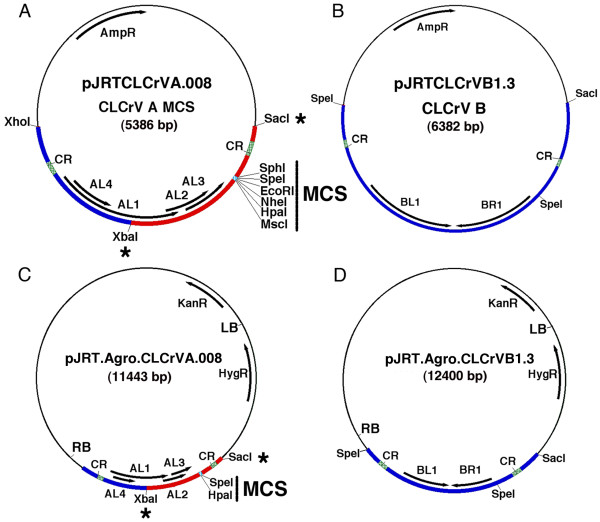
**Details of the CLCrV-derived VIGS vectors described in this paper. A, B)** Maps of CLCrV plasmids for biolistic inoculation. **C,D)** Maps of the CLCrV:CP- (C) and CLCrVB (D) binary vectors for agroinoculation. The pBluescript SKII + (A,B) and the pCambia1300 (C,D) plasmid backbones are shown by a thin black line and geminivirus DNA is blue or red. Unique *Sac*I and *Xba*I sites that may be used to sub-clone silencing fragments from the biolistic vector into the *Agrobacterium * vector are marked with asterisks, and the resulting fragment is shown in red. MCS – multiple cloning site (light blue), CR – common region (green), LB and RB – left and right T-DNA borders respectively, HygR – hygromycin resistance, AmpR – ampicillin resistance, KanR – kanamycin resistance. AL1, AL2, AL3, and AL4 are genes encoded by the A-DNA and BL1 and BR1 by the B-DNA of CLCrV.

Many labs lack the necessary equipment to inoculate the biolistic form of the CLCrV vector. To address this, we cloned each of the vector's components into the open-source binary plasmid, pCambia1300 (CAMBIA, Canberra, Australia). The A-DNA vector, pJRT.Agro.CLCrVA.008 (referred to as CLCrVA:CP-), contains a multiple cloning site in place of the coat protein gene and produces an episome that is identical to that from the biolistic vector following inoculation. Episomes from the B-DNA binary plasmid, pJRT.Agro.CLCrVB1.3 (referred to as CLCrVB), are also identical to their biolistic counterpart. Two additional A-DNA plasmids, one for silencing *ChlI* (pJRT.Agro.CLCrVA.009) and one expressing GFP (pJRT.Agro.CLCrVA.010), were made by swapping *XbaI*/*SacI* fragments consisting of the 3' region of the *AL1* gene, *AL2, AL3*, the multiple cloning site, and one of the two duplicated common regions with the same region from the biolistic vector (Figure 1). These plasmids will be referred to as CLCrVA:ChlI and CLCrVA:GFP, respectively. Binary plasmids were transformed into the *Agrobacterium *strain GV3101:pMP90 [26] using the freeze-thaw method [27]. The plasmid pMP90 was derived from the pTiC58 plasmid pGV2201 and contains a deleted T-DNA region, the necessary virulence functions, and gentamycin resistance [26].

**Figure 2:**
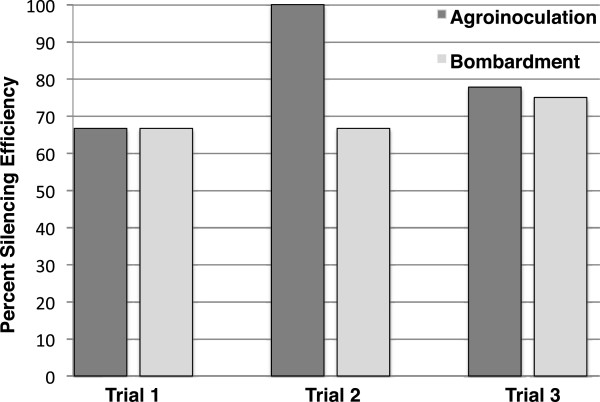
**The efficiency of silencing from agroinoculation and particle bombardment of CLCrVA:ChlI was comparable.** Dark and light grey bars represent silencing efficiencies in three agroinoculation or three particle bombardment experiments, respectively. Particle bombardment was conducted as described previously (12) with the homemade gun described by Finer and coworkers (15).

**Figure 3:**
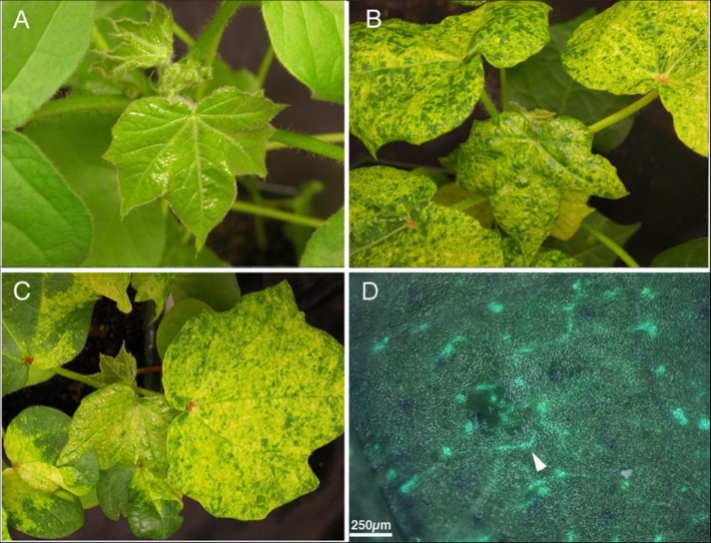
**Silencing and expression from the CLCrV  * Agrobacterium * vector was comparable to the biolistic vector. A)** New growth on cotton plants agro-inoculated with the CLCrVB and CLCrV:CP- lacked visible symptoms at 35 dpi. **B)** New growth on cotton plants agro-inoculated with CLCrVB and CLCrVA:ChlI showed a sectored loss of chlorophyll throughout the leaf at 35 dpi. **C)** Plant bombarded with the biolistic form of the vector used in (B) showed a similar pattern of silencing as the plant shown in (B) at 38 dpi. **D)** Overlay of GFP and brightfield images showed vein-delimited GFP expression (white arrowhead) in new growth of plants agro-inoculated with CLCrVB and CLCrVA:*GFP* at 28 dpi.

We used CLCrVA:ChlI to compare the efficiency and extent of silencing from the biolistic and *Agrobacterium- *based methods of delivery. Cotton seedlings were agroinoculated by infiltration of the bottom surface of the cotyledons with a 1-ml syringe lacking a needle. The inoculum consisted of 1:1 mixtures of *Agrobacterium * cultures harboring CLCrVA:ChlI or CLCrVB. Following infiltration, the*ChlI* silencing phenotype of leaf yellowing was first observed at 12 to 20 days post inoculation (dpi). Both particle bombardment and agroinoculation methods resulted in similar silencing efficiencies (Figure 2).We obtained an average inoculation efficiency of 81% (n = 30) over three agroinoculation experiments, which was not significantly different from the 69% efficiency we observed for three biolistic experiments (n = 27; p-value 0.34; two-tailed non-parametric t-test). Both the onset and extent of *ChlI* silencing after agroinoculation were similar to plants inoculated by particle bombardment (Figure 3). Similarly, the expression of soluble-modified red-shifted GFP (smRS-GFP) [28] from the *Agrobacterium *vector CLCrVA:GFP was also comparable to particle bombardment. As described previously [12], GFP fluorescence was confined to vascular associated cells, reflecting the known tissue specificity of CLCrV (data not shown).

The biolistic vector has 6 different unique restriction sites in its MCS but, due to its larger size, only two of these sites are unique in the *Agrobacterium * vector. This makes cloning silencing fragments into the *Agrobacterium *vector more difficult. To overcome this, silencing fragments can be introduced into the biolistic CLCrV A vector and then sub-cloned into the *Agrobacterium * vector using *Sac*I and *Xba*I (Figure 1). Although not in the MCS, the *Sac*I and *Xba*I sites occur only once in both of the CLCrV A vectors.

One minor drawback of agroinoculation was that initial seedling growth was slightly delayed compared to their biolistic counterparts for both *ChlI* silencing and GFP expression constructs. This difference occurred despite the production of identical viral episomes in both cases. The transient slower growth of agroinoculated plants was consistent with native *Agrobacterium *acting as a plant pathogen [29] and causing systemic changes in inoculated plants [30].

### Development of low-cost particle inflow guns

**Figure 4:**
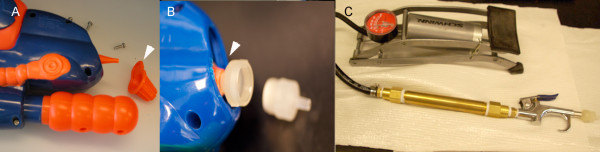
**Examples of two homemade gene guns**. **A, B)** A plastic gene gun made from a slightly modified 5-dollar water gun. **A)** The only modification required was to connect a Millipore swinex filter tip to the nozzle for compressed air. This was achieved by removing three screws in the plastic body, followed by the plastic nozzle cover (arrow). **B)** The $5 gene gun with the filter tip screwed into the cut-off nozzle (arrow). **C)** An example of a metal gene gun built for approximately $50 from parts at a local hardware store (see Materials and Methods for construction details).

As an alternative to commercial particle bombardment systems, a 5-dollar pump-action plastic water gun can serve as a gene gun (Figure 4A,B). The only modifications required were the removal of a small plastic tip covering the outlet nozzle, and cutting of the outlet nozzle to match the outer diameter of a Millipore swinex syringe filter, used to hold the DNA-coated particles (Figure 4B). The gun employed a small hand-powered pump to charge a cylinder with compressed air, which was then released to travel through the filter tip so that the 1-micron gold particles carrying the VIGS vectors were propelled into the target cells. At least one comparatively simple gene gun has been described previously [17], but required the use of a separate compressed air supply.

**Figure 5:**
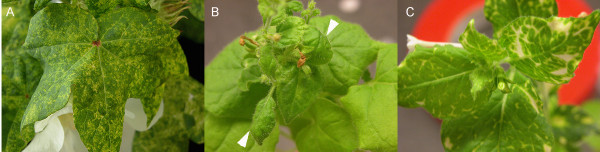
** *ChlI*  silencing from two different VIGS vectors inoculated using two low-cost particle inflow guns. A)***ChlI* silencing in cotton (64 dpi) co-bombarded with the CLCrVA:ChlI and CLCrVB plasmids using the modified plastic water gun shown in Figure 4A,B. **B)***N. benthamiana* (37 dpi) showing the viral symptom of contorted upper leaves (arrows) after bombardment with the wild type TGMV virus using the homemade metal gene gun. **C)***N. benthamiana* (37 dpi) showing *ChlI* silencing after co-bombardment with TGMVA and TGMVB:ChlI using the metal gene gun (Figure 4C).

This gun was used to inoculate cotton with the biolistic CLCrV-based VIGS vector carrying a 500-bp fragment of the *ChlI* gene. The number of pump strokes was used to gauge pressure in the gun, and 40 to 60 strokes resulted in successful inoculation of the CLCrV silencing vector. Inoculation efficiency was visually assessed by counting the number of plants showing photobleaching. In a preliminary experiment, silencing was observed in 1 of 3 inoculated cotton plants, but in a subsequent experiment silencing was observed in 3 of 6 inoculated cotton plants (Figure 5A). More plants can be inoculated to compensate for the relatively low inoculation efficiency with this simple gene gun. The extent of silencing (yellow leaf area) was similar to plants inoculated by other methods.

Although it was useful, the modified water gun did not withstand the rigorous cleaning needed to conduct sterile bombardments. Therefore, we developed a more durable version of the gun from off-the-shelf parts purchased at a hardware store for approximately 50 dollars. Most of the cost was attributable to the brass components. The metal gun was used to introduce principles of gene silencing to students in a biochemistry lab. In these experiments both wild type and *Tomato golden mosaic virus* (TGMV)-derived VIGS vectors were inoculated into *Nicotian abenthamiana.* Efficiency was assessed with either photobleaching from the TGMV silencing vector carrying a 156-bp *ChlI* fragment (TGMVB:ChlI [9]) or symptom development (contorted leaves) from the wildtype virus. The built-in gauge on the bicycle pump was used to measure pressure in the gun. A pressure of 80 lb/in^2^ was sufficient to obtain silencing in 3 of 5 inoculated plants and to produce TGMV infection in 5 of 5 inoculated plants (Figure 5B,C).

## Conclusions

Tools for several affordable methods for the delivery of the CLCrV VIGS vector have been developed and demonstrated. The new molecular tools were binary plasmids for agroinoculation of the CLCrV cotton VIGS vector by syringe infiltration. Agroinoculation resulted in essentially equivalent efficiency and extent of silencing compared to biolistic inoculation. The *Agrobacterium *vectors were larger than the biolistic VIGS vectors and thus contain fewer unique restriction sites for cloning in silencing fragments, but the ability to sub-clone fragments from the biolistic vectors circumvents this problem. The new hardware consisted of two low-cost ($5 - $50) particle inflow gunsmade exclusively from off-the-shelf components that can be used instead of expensive commercial devices for biolistic inoculation. The $50 gene gun is durable and withstands rigorous cleaning while also being easy to construct and use. These simple and affordable biolistic guns will extend the potential for research and teaching based on VIGS.

## Methods

### Vector availability

All CLCrV vectors described here are available for non-profit research from Addgene.org following a standard material transfer agreement.

### Plant material

All cotton plants (*G. hirsutum* cv Deltapine 4515) were grown in a 25°C/23°C (day/night) chamber at approximately 60% relative humidity under a bank of 16 VHO T5 fluorescent lamps fixed at a height of 27 inches. Lighting (135 μmol s^-1^ m^-2^ at the shelf level) was long-day: 16 hours on, 8 hours off. Cotton plants were grown in 6-in diameter pots in Metromix 360 potting mix (Wyatt Quarles Seed Company, http://www.wqseeds.com), watered daily, and fertilized once per week with Miracle Grow® (The Scotts Company LLC, http://www.scotts.com).

### Cloning the vector into pCambia

To generate pJRT.Agro.CLCrVA.008, the biolistic empty vector plasmid pJRTCLCrVA.008 [12] was digested with *Xba*I and *Xho*I. A 1130-bp fragment from pJRTCLCrVA.008 containing one common region, AL4, and the 5' 664-bp of AL1 was ligated into pCambia1300 cut with *Xba*I and *Sal*I to produce pJRT.Agro.CLCrVA.008.1. In a second digestion and ligation, pJRTCLCrVA.008 was cut with *Xba*I and *Sac*I and the 1386-bp resulting fragment was cloned into pJRT.Agro.CLCrVA.008.1 cut with *Xba*I and *Sac*I to generate a 11,443-bp plasmid pJRT.Agro.CLCrVA.008.

To produce the *ChlI-*silencing and GFP-expressing marker constructs pJRT.Agro.CLCrVA.009 and pJRT.Agro.CLCrVA.010, the biolistic constructs pJRTCLCrVA.009 and pJRTCLCrVA.010 were each digested with *Sac*I and *Xba*I to release 1,883-bp and 2,062-bp fragments respectively. These fragments were then ligated into *Sac*I, *Xba*I digested pJRT.Agro.CLCrVA.008 to produce pJRT.Agro.CLCrVA.009 and pJRT.Agro.CLCrVA.010.

Prior to inserting the B component of the viral genome into pCambia1300, it was necessary to reduce the duplication of B component sequence from a complete dimer [31] to a version with only one BR1 gene. This was accomplished by using PCR and the primers BDAgR1new (5’ – ACC CAG ACT AGT AAA CGC TAT TAT ATA GG – 3’) and BDAgF1 (5’ - GCG GAG CTC CAG AAC GAT CTC AGT TAG GTC ATG GG - 3’) to amplify a 906-bp fragment, containing a single common region, from the CLCrV B dimer. The BDAgF1 primer introduced a *Sac*I restriction enzyme site at the 5’ end of the PCR product. The additional *Sac*I site was used in conjunction with an internal *Spe*I site to insert the 896-bp fragment into the respective restriction sites of the pBluescript SK + II vector (Agilent Technologies, http://www.agilent.com) to create pJRTCLCrVB.1. Next, a 2,549-bp fragment was cut from the CLCrV B dimer using the restriction enzyme *Spe*I. This fragment contained a second viral common region as well as the two full-length open reading frames *BL1* and *BR1*. The fragment was purified by gel extraction and ligated into the *Spe*I restriction site of pJRTCLCrVB.1. The resulting clones were screened for proper orientation of the insert by *Xba*I digestion and called pJRTCLCrVB1.3. The plasmid JRTCLCrVB1.3 contains two viral common regions that flank the two full-length open reading frames *BL1* and *BR1* in the pBluescript SK+ II vector.

After construction of pJRTCLCrVB1.3, the construct was digested with the restriction enzymes *Hind*III and *Sac*I. The 3,490-bp fragment produced from this digestion was cloned into the respective sites in the *Hind*III*/Sac*I digested and dephosphorylated pCambia1300 plasmid to produce the 12,400-bp plasmid pJRT.Agro.CLCrVB1.3.

### Agroinoculation of the vector

*A. tumefaciens *strain GV3101:pMP90 was first made competent by inoculating 50 ml of Luria Bertani medium with 125 μL of an overnight *Agrobacterium * culture. The 50-ml culture was then grown at 30°C for 12 hours, spun for 10 minutes at 4000 x rpm to pellet cells, and washed in 5 ml of sterile TE buffer; this step was repeated once. The TE buffer was removed and the cells were then resuspended in 5 ml of Luria Bertani medium and 200-μL aliquots were refrozen in liquid nitrogen.

For transformation, cell aliquots were thawed on ice, and 1 μg of plasmid DNA was added to the cells on ice for 5 minutes. Next, the cells and DNA were transferred to liquid nitrogen for 5 minutes, then a 37°C water bath for 5 minutes. 1 ml of Luria Bertani medium was added to the tube, and the cells were incubated at room temperature for 4 hours with agitation. The culture was spun down for 10 minutes at 4000 rpm and all but approximately 100 μL of the supernatant was removed. Cells were resuspended and then plated on Petri plates with 25 μg/ml each of kanamycin, rifampicin, and gentamycin and allowed to grow for 2 days at 30°C.

The vector was introduced into cotton seedlings by infiltration. Cultures of each component were prepared as previously described [32]. After adjusting the density of each culture (OD_600 _of 1.5) and allowing them to incubate at room temperature in the dark for 4 hours without shaking, A and B component cultures were mixed in a 1:1 ratio and drawn up into a needleless 5-cc syringe. The mixture was introduced into cotton seedlings at the cotyledon stage by gently forcing the solution into the spongy mesophyll on the bottom surface of the cotyledon. As the infiltration solution moved through the cotyledon, it became noticeably darker, and infiltration was continued until the entire cotyledon was darkened.

### Construction of a low-cost particle delivery device

To construct an extremely low cost particle delivery device, we first purchased a pump-action water gun; the MAX D 2000 (Hasbro Inc., Hasbrotoyspr@hasbro.com) from a local store for approximately 4 dollars. A small Phillips head screwdriver was used to remove three screws from the plastic housing on the front of the gun. This allowed the plastic housing to be pried open just enough for the plastic nozzle cover to be removed. The exposed conical nozzle of the gun was then modified to hold a 13 mm Millipore swinex filter tip (Millipore Inc., http://www.millipore.com). The plastic nozzle was cut back (~18 mm) to the outer housing so that its inner diameter was large enough to allow for the insertion of the threaded filter tip. For inoculation, the built-in hand pump was pumped 40–60 times immediately prior to bombardment. No vacuum was used and the tip of the Millipore filter was placed less than a centimeter from the bottom surface of the cotyledon.

A second more durable gun was constructed consisting of a bicycle pump connected to one end of a brass cylinder via a section of 6-ft fuel line with a ¼-in inner diameter. A blowgun was adapted to the other end of the brass cylinder and fitted with a 13-mm Millipore swinex tip. A bicycle pump with a pressure gauge rated up to 100 lb/in^2 ^(Schwinn Inc., http://www.schwinn.com) was adapted to the fuel line using a ¼-in inflation nozzle (provided in a Kobalt 5-PC blowgun kit; Lowes Inc., http://www.lowes.com, Item #001174).This was essentially a ¼-in threaded male fitting on one end and a barb on the other. The fuel line was secured to the barb using a #4 pipe clamp (King Seal Fastener Technology Co., Ltd., Anhui, China, Item #62508). The other end of the fuel line was connected to a brass ¼-in male barb adapter (Watts Industries Inc., http://www.watts.com, Item #A-192) and was secured with a #4 pipe clamp. The ¼-in male barb adapter was screwed into a female ¼-in to ½-in brass coupling (Watts Industries Inc., Item #A-813) that was threaded onto a ½-in X 6-in brass pipe nipple (Watts Industries Inc., Item #LA-845). Another ¼-in to ½-in female coupling was put in place at the other end of the brass pipe. This coupling was attached to a Kobalt blowgun via a ¼-in male brass nipple (Watts Industries Inc., Item #A-875). The end of the blowgun was fitted with a blowgun adapter included in the above mentioned kit, and the Millipore swinex filter tip was threaded directly into this adapter. For inoculation of *N. benthamiana* with TGMV, the bicycle pump was pumped to approximately 80 lb/in^2^ and the trigger of the blowgun was quickly compressed with the tip of the Millipore syringe filter held less than 1 cm from the surface of the leaf.

For a schematic image of this gene gun see Additional file 1: Figure S1.

### Microcarrier preparation

For particle bombardment, aliquots of 1 μm gold particles (INBIO Gold, Victoria, AU, Catalog #BD061) were prepared by suspending 60 mg of gold powder in 1 ml of 100% ethanol. Particles were pelleted by centrifugation for 10 seconds at 13,000 rpm. The supernatant was discarded and the particles were resuspended in 1 ml of sterile water. The particles were pelleted by centrifugation and resuspended in sterile water two additional times. Following the final resuspension, 50-μl aliquots were transferred to 1.5 ml microfuge tubes and stored at −20°C.

To precipitate viral DNA onto the gold particles, 5 μg of each viral component was added to a 50-μl aliquot and vortexed for 30 seconds. 50 μl of 2.5 M CaCl_2_ was added and the tube was vortexed for 30 seconds. 20 μl of 0.1 M spermidine was added to the tube and the mixture was vortexed for 3 minutes. The particles were pelleted by centrifugation at 10,000 rpm for 10 seconds. The supernatant was removed and discarded. The pellet was resuspended in 250 μl of 100% ethanol by vortexing. The particles were again pelleted by centrifugation at 10,000 rpm for 10 seconds and the supernatant was discarded. The pellet was resuspended in 65 μl of 100% ethanol. 12 μl of the particle suspension was loaded onto the center of the filter tip for bombardment (see Additional file 2: Figure S2).

## Abbreviations

VIGS, Virus-induced gene silencing; CLCrV, Cotton leaf crumple virus; TGMV, Tomato golden mosaic virus; PTGS, Post-transcriptional gene silencing; PIG, Particle inflow gun; ChlI, Magnesium chelatase subunit I; dpi, Days post infection; smRS-GFP, Soluble-modified red-shifted green fluorescent protein; CR, Common region; LB, Left border; RB, Right border.

## Competing interests

The authors declare that they have no competing interests.

## Authors’ contributions

JRT carried out the molecular cloning, test inoculations, developed the gene guns, and drafted the manuscript. CHH and DR helped to plan research and write the manuscript. All authors read and approved the final manuscript.

## Supplementary Material

Additional file 1 ** Schematic image of metal gene gun.** Image of a disassembled metal gene gun labeled with corresponding part numbers. Click here for file

Additional file 2 ** Close-up image of Millipore swinex filter tip.** Close-up image of a disassembled Millipore swinex filter tip. The red arrow marks the placement of the microcarrier suspension. Click here for file
