# Supplementary figures and images for: Method: low-cost delivery of the cotton leaf crumple virus-induced gene silencing system
Source: Plant Methods. 2012 Aug 1;8:27. doi: 10.1186/1746-4811-8-27 (PMC3441267; doi:10.1186/1746-4811-8-27)

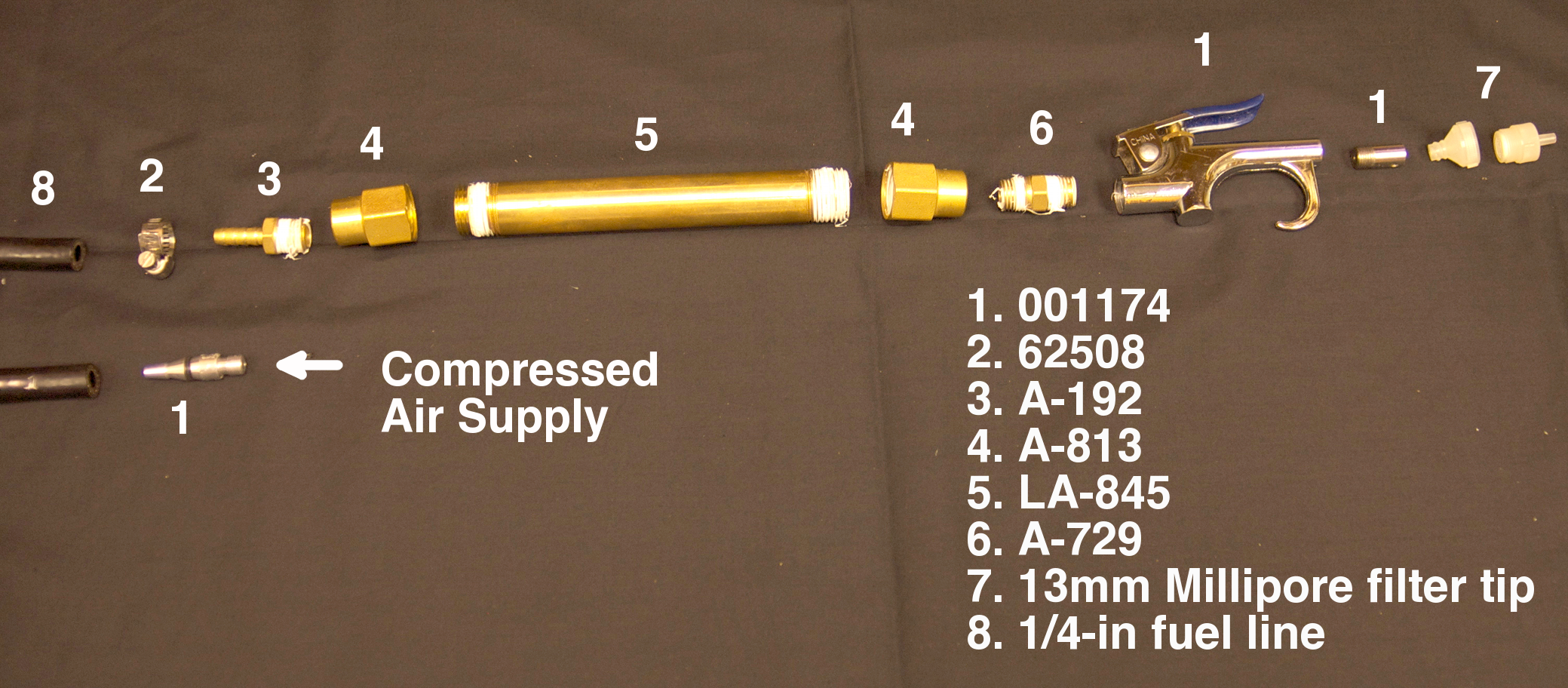

Supplement: Additional file 1 — Schematic image of metal gene gun. Image of a disassembled metal gene gun labeled with corresponding part numbers. [file 1746-4811-8-27-S1.tiff]

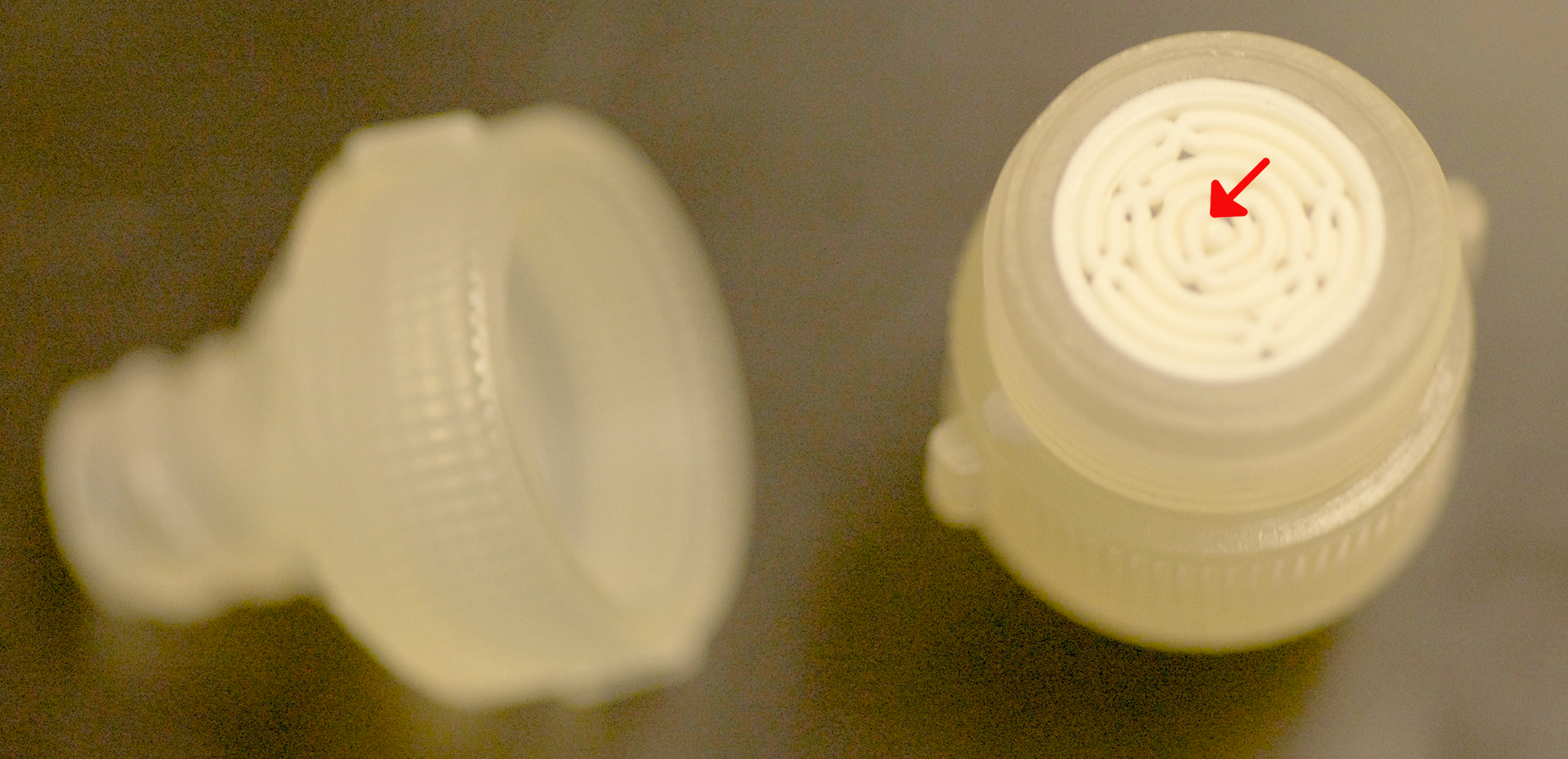

Supplement: Additional file 2 — Close-up image of Millipore swinex filter tip. Close-up image of a disassembled Millipore swinex filter tip. The red arrow marks the placement of the microcarrier suspension. [file 1746-4811-8-27-S2.tiff]
